# Communicative deficits associated with maladaptive behavior in individuals with deafness and special needs

**DOI:** 10.3389/fpsyt.2022.944719

**Published:** 2022-07-29

**Authors:** Johannes Fellinger, Magdalena Dall, Christoph Weber, Daniel Holzinger

**Affiliations:** ^1^Research Institute for Developmental Medicine, Johannes Kepler University of Linz, Linz, Austria; ^2^Institute of Neurology of Senses and Language, Hospital of St. John of God, Linz, Austria; ^3^Department of Psychiatry and Psychotherapy, Clinical Division of Social Psychiatry, Medical University of Vienna, Vienna, Austria; ^4^Department for Inclusive Education, University of Education Upper Austria, Linz, Austria; ^5^Institute of Linguistics, Faculty of Humanities, University of Graz, Graz, Austria

**Keywords:** deaf, intellectual disabilities, social communication, language skills, maladaptive behavior

## Abstract

**Background:**

At least one in three individuals who are prelingually deaf has special needs, most commonly due to intellectual disabilities. The scant literature on challenging behavior in this population, however, suggests high rates of prevalence and an important need to better understand the contributing factors.

**Aim:**

We sought to analyze the prevalence of maladaptive behavior and its association with intellectual functioning, adaptive skills, language skills, and social communication in a population of adults with deafness and special needs.

**Methods:**

Participants were 61 individuals from three therapeutic living communities established for people with deafness and special needs. The participants had a mean age of 54.7 years, 64% were male. Intellectual functioning was measured with two versions of the Snijders–Oomen Non-verbal Intelligence Scale. The Vineland-II Scales were used to assess adaptive and maladaptive behavior. Language skills were measured with instruments specifically adapted for this population, including the Reynell Developmental Language Comprehension Scale, the comprehension scale of the Child Development Inventory, and the Profile of Multiple Language Proficiencies. Due to high correlations between instruments, a composite language score was used. A specific questionnaire to measure social communication in adults with intellectual disabilities was also utilized.

**Results:**

The mean nonverbal developmental reference age was 6.5 years, whereas the equivalent for the language measures was about 3.5 years. The prevalence rate of elevated maladaptive behavior was 41% (v-scale score ≥18) and 18% of the participants had a clinically significant score (v-scale score ≥21). Regression analyses showed that only language and social communication skills were significantly associated with maladaptive behavior, while intellectual functioning and adaptive skills were not.

**Conclusion:**

These findings emphasize the importance of the constant promotion of communicative skills, as those people with better language and social communication skills demonstrate lower levels of maladaptive behavior.

## Introduction

Prevalence rates of hearing loss range between 15 and 25% in the adult population. Hearing loss with onset before language acquisition with a prevalence rate of about 2 per thousand can have a tremendous impact on communication and social-emotional and cognitive development (1). Particularly the first years of life are critical for language and general development. If a child does not get sufficient access to spoken or signed language during that important time period, this can have longlasting effects on the future ability of learning a language and this language deprivation and consequently social isolation can severely affect mental health later in life (2–4). Approximately 33–50% of individuals who are prelingually deaf or hard-of-hearing have additional disabilities (5, 6), most commonly intellectual disability (ID) (7), other neurodevelopmental disorders such as Autism Spectrum Disorder or Attention Deficit Hyperactivity Disorder, dual-sensory impairment (deaf-blindness), and neurological disorders (e.g., epilepsy, cerebral palsy), with frequent comorbid psychiatric conditions. In this population, sometimes referred to as “Deafplus” or “Deaf+” (8), communication and mental health difficulties are even more pronounced. In the literature the combination of deafness and intellectual disability is refered to as a “double jeopardy,” indicating that the occurrence of both deafness and intellectual disability together has a much greater impact on people's lives than the sum of the two (9, 10).

The current literature shows that children and adolescents who are deaf have a higher risk of emotional and behavioral difficulties (11). In the adult population with prelingual deafness, the rates of mental health problems are higher than those of the population without deafness (12–16). In a Danish study, children with both deafness and special needs had a three times higher rate of psycho-social problems compared to children with deafness but without additional needs (17).

The literature on the mental health of adults who are deaf and have special needs is very limited. Timehin and Timehin (10) studied a large sample of residents in homes for people with learning disabilities and found problematic behavior in 62% of the individuals who had hearing impairments as well as a learning disability, which is dramatically higher than the 10–15% prevalence rate reported for individuals with intellectual disabilities in general (18, 19). Buskermolen and colleagues (20) observed 21 individuals with deafness and intellectual disabilities for 1 year, and found that in this small population, challenging behavior was present 25% of the time (ranging widely from only 1.8 to 75.4% of the time).

The emotional and behavioral problems of deaf children and the associations with language problems are well documented in the literature (11, 21–23). The same is also true of people with only ID. In general, communication impairments are common correlates of challenging behavior (19). In the study by Buskermolen and colleagues (20), individuals who had higher levels of communication and were socially more independent showed challenging behavior on fewer occasions. However, the instruments used to measure social independence and communicative development in this study were not stated and the sample was very small.

The key role of communication in the development of neurocognitive processes, including inhibition and attention and also the learning of adaptive behavior and social norms, has been evident for a long time (24). The population-based longitudinal ALSPAC study (25) examined associations between social communication skills and trajectories of conduct problems through childhood to adolescence (4–13 years). Four conduct-problem pathways were identified: early-onset persistent (9.0%), childhood-limited (9.0%), adolescent-onset (14.7%), and low (64.6%). Social communication problems were significantly greater in all conduct-problem groups, with deficits in the early-onset-persistent group especially marked. The correlations between communication difficulties and conduct disorders remained robust after controlling for demographic confounders and verbal IQ.

However, research data about the highly vulnerable population of people who are deaf with special needs (mainly ID) are lacking. More recently, however, the role of social communication, which is the appropriate use and understanding of verbal and non-verbal language in social interactions, is gaining attention as a more predictive element of psychosocial functioning than structural language (vocabulary and morphosyntax) (26). Social communication encompasses the whole variety of communicative functions (such as greeting, making requests, or asking for information), conversational skills (such as taking turns, communication repair strategies, or staying on topic), and the adaptation of communication to the interlocutor and situation (e.g., meeting the interests of the interlocutor or ensuring politeness). In children, studies have shown associations between conduct problems and difficulties in pragmatic language (27, 28).

In the literature, “maladaptive behavior… is defined as behavior that interferes with an individual's activities of daily living or ability to adjust to and participate in particular settings” (29). Using this definition, we conducted an analysis of the prevalence of maladaptive behavior and its association with language skills and social communication in adults with deafness and special needs. We hypothesized that primarily language skills would be negatively associated with maladaptive behavior and that social communication skills would have an even higher prediction than structural language skills for maladaptive behavior and that other associations such as intellectual functioning have a weaker influence within this deaf+ population.

## Materials and methods

### Therapeutic living communities

This cross-sectional study was conducted in three therapeutic living communities for people with deafness and an ID and/or other neurodevelopmental or psychiatric disorder(s), in Upper and Lower Austria. The communities in which participants were recruited are characterized by the constant use of visual communication (signed language). All staff members use sign language, which is adapted to the communication skills and needs of the participants. Around 25% of the staff are deaf themselves, and all staff members are fluent in sign language. Furthermore, other forms of visual communication, such as pictograms are used. There is a strong focus on the constant development of communicative skills in daily life. The staff is trained in principles of participant-guided communication, leaving the initative and control of conversations to the interlocutor and at the same time creating situations that stimulate communication. One of the main goals is the use of facilitative language techniques adapted to the individuals language and communication level within natural everyday situations, from low-level techniques such as labelling objects and activities in the participant's current focus of interest, to higher level techniques such as expanding the participants utterances or asking open questions that invite for elaborated communication. Most participants live and work in the communities. A minority of individuals only attend the day workshops, and these offer woodworking, cooking, textiles, and pottery.

Data collection took place between 2017 and 2020. All variables (intellectual functioning, adaptive and maladaptive behavior, language, and communication skills) are routinely assessed for every participant in the therapeutic living community. Intellectual functioning was directly assessed by the staff psychologist, whereas adaptive and maladaptive behavior was assessed together with either a family member or a staff member acting as the primary caregiver. Language skills were directly assessed by a linguist and proxy-rated by caregivers, and social communication skills were assessed by caregiver observations.

The study was approved by the ethical committee of the [Hospital St. John of God in Linz]. Consent for participation was given by the participants themselves and/or by their legal guardian (if applicable).

### Instruments

#### Intellectual functioning

Due to the different intellectual levels of participants ranging from borderline intellectual functioning to severe ID, two versions of the Snijders–Oomen Non-verbal Intelligence Scale for individuals were used: SON-R 6-40 (25) and SON-R 2 $\frac{1}{2}$-7 (30). Since the SON-R 2 $\frac{1}{2}$-7 does not provide an IQ score, the reference age of intellectual functioning was used for all participants.

#### Adaptive and maladaptive behavior

The Vineland-II Scales (31) are a well-established and recognized instrument to measure adaptive behavior. The standardized, structured interview is norm-referenced for individuals from birth to 90 years of age. For the participants of this study, the interview was conducted by the staff psychologist of the therapeutic living communities with either close relatives of the participants where possible, or with each participant's primary caregiver at the therapeutic living community. The dimensions investigated were motor, communication, daily living, and social skills. For each domain, a standard score (M = 100, SD = 15) can be calculated. An adaptive behavior composite score including all domains can also be calculated. However, the communication domain refers to, among other things, the comprehension and production of spoken language. Even after adjusting the items to the visual modality and sign language items that measure a similar level of complexity, there was still a strong floor effect in the communication dimension in our sample. Therefore, the language and communication skills of the participants were assessed with more appropriate instruments for this target population. For our analysis, we computed an adaptive skills composite score covering social skills, daily living skills, and motor skills (Cronbach's alpha = 0.84).

Maladaptive behavior was also assessed *via* three dimensions: internalizing, externalizing, and other. In addition, an overall maladaptive behavior index was calculated and used in the present study. The internalizing subscale includes questions such as: “is overly dependent,” “is overly anxious or nervous,” and “avoids social interaction.” Examples of the questions for the externalizing subscale are “is impulsive,” “taunts, teases, or bullies,” and “behaves inappropriately at the urging of others.” The third subscale titled “other” includes items such as: “is overly familiar with strangers,” “has a hard time paying attention,” and “ignores or doesn't pay attention to others around him/her.” The outcome of the internalizing, externalizing, and maladaptive behavior index scales is grouped into three categories according to the v-scale score: average (<18), elevated 18–20, or clinically significant 21–24. An elevated score is equivalent to having higher levels of maladaptive behavior than 84% of individuals in the normative sample at the same age. A clinically significant score means that the individual is within the highest 2% of the standardization sample regarding maladaptive behavior (31). The internal consistency (Cronbach's alpha) of the internalizing, externalizing, and overall maladaptive scale for the age range 40–90 years ranges between 0.67 and 0.85 (31).

#### Language and social communication

Due to the lack of measurements of visual communication and signed language skills for individuals with ID communicating visually, two standardized assessments for spoken language were adapted and tested in pilot studies. The language comprehension scale of the Reynell Developmental Language Scale-III (RDLS-III) (32) provides norms for spoken English for the age range of 1;10–6;11 years. The scale was adapted to Austrian Sign Language by hearing and deaf linguists, speech and language therapists, and teachers of the deaf (33). The adapted RDLS-III measures *sign language comprehension* of increasingly grammatically complex utterances through direct assessment. It consists of 62 items, including 15 single signs and 47 sign-language utterances that are presented by fluent signers and responded to by the participants by acting out or pointing at pictures (play materials and four-field tables). The adaptation has so far been tested only with a small sample of 10 children born to deaf parents (aged between 1;10 and 9;7 years) growing up with Austrian Sign Language as their first language. This study demonstrated high correlations between the raw scores and participant age as well as non-verbal cognitive age. Furthermore, high correlations with parental assessments of language comprehension were found. The measure can therefore be regarded as promising for use in the assessment of language comprehension in signing populations (33). Because of the lack of normative data, we used raw scores to investigate correlations between sign language comprehension and behavior in this study.

Another instrument to assess *sign-language comprehension* is the adapted version of the Comprehension Scale of the Child Development Inventory (CDI) (34) which measures sign language comprehension by caregiver report. The measure includes 50 items ordered by increasing complexity. In most cases, the translation and adaptation of the language samples into Austrian signs and sign language structures was possible. Eight items had to be replaced because they were not directly translatable into visual communication. For this purpose, we followed the Visual Communication and Sign Language Checklist for signing children (35). The adapted version was used in the same sample of 10 children growing up with Austrian Sign Language as the family language reported above (33). The high correlation between the raw scores and children's age (r = 0.743, *p* = 0.014) and the direct assessment of language comprehension (adapted RDLS-III) (*r* = 0.868, *p* = 0.001) suggest good validity of the measure.

Two parallel eight-level scales of the Profile of Multiple Language Proficiencies (PMLP) (36) were used by caregivers to *approximate expressive and receptive language skills* in American Sign language and spoken English. Originally developed for American Sign Language, the sign language scales were adapted to Austrian Sign Language by one of the authors [DH]. The stages range from pre-linguistic levels, word-combinations, use of simple and compound sentences, up to complex sentences to full fluency, and are described with reference to vocabulary, morphosyntax, and functional language. The raters are asked to select the level for which an individual fulfills most of its criteria.

To reduce the number of language-related measures (described above), we conducted principal component analysis and found a dominant component with an Eigenvalue of 3.5 (87% explained variance). Thus, we computed a language skills composite score (Cronbach's alpha = 0.75) to be used in the analyses.

The social communication questionnaire in adults with ID (QSC-ID) (37) was developed to collect data on five aspects of social communication that are assumed to be strongly interrelated: (i) an individual's engagement in social communication, (ii) conversational skills, (iii) adaptation of communicative behaviors to an interlocutor, (iv) pro-social use of communication, and (v) use of non-verbal communication. The QSC-ID is a proxy measure to be used by caregivers to collect information on functional communication skills that need to be considered in planning interventions. The QSC-ID provides a five-point Likert scale ranging from “applies fully” to “does not apply at all” (37). In a pilot study with 52 deaf adults with ID, high construct validity (high correlations with social skills, language, and autism symptom scores, and moderate correlations with adaptive skills and nonverbal cognition) were found. Internal consistency was excellent (Cronbach's alpha 0.93–0.96) and interrater reliability between caregivers in working and living environments was good (ICC = 0.80).

### Participants

The sample consisted of 61 participants who were deaf or hard of hearing with intellectual disability and/or other neurodevelopmental or psychiatric disorders and living or working in one of three therapeutic living communities. See Table 1 for the sample description. All participants have in common, that before the age of 6 years, when they got enrolled in a school for the deaf, they had almost no access to sign language. Due to the lack of newborn hearing screening and fitting of effective hearing technology when the participants were at pre-school age, auditory access to spoken language was insufficient. As a consequence, they grew up without adequate access to language and subsequent minimal expressive language. In most families, only a limited number of simple home signs and gestures were used, leading to a severe language deprivation during childhood. All participants were born to hearing parents and many grew up decades ago in rural areas, without any possible contact to other people who were deaf. The mean age was 54.74 (SD = 18.56) years and 63.9% of participants were male. The large majority (85.2%) had profound hearing loss and almost 50% had moderate to severe ID. The mean non-verbal cognitive functioning reference age was 6.23 (SD = 2.77) years. Around 25% of the participants were diagnosed with epilepsy and almost as many (21.7%) had cerebral palsy. There were 9 participants (15%) on the autism spectrum.

**Table 1:** Sample description (*N* = 61).

**Characteristics**	***N*** = **61**
Age, years M (SD)	54.74 (18.56)
Sex male *n* (%)	39 (63.9)
Non-verbal cognitive functioning reference age M, years (SD)	6.23 (2.77)
Non-verbal cognitive functioning reference age *n* (%)	
*>9 years*	9 (14.8)
*6–9 years*	24 (39.3)
*3–6 years*	25 (41.0)
* <3 years*	3 (4.9)
Degree of hearing loss *n* (%)	
*Moderate hearing loss (40–69 dB)*	2 (3.3)
*Severe hearing loss (70–89 dB)*	7 (11.5)
*Profound hearing loss (>90 dB)*	52 (85.2)
Autism spectrum disorder *n* (%)	9 (15.0)
Epilepsy *n* (%)	16 (26.7)
Cerebral palsy *n* (%)	13 (21.7)

### Analyses

First, we calculated correlations between the set of independent variables and the outcome of maladaptive behavior. Second, we used regression analysis to evaluate the independent contribution of adaptive skills, language skills, and social communication to maladaptive behavior. Notably, due to the small sample size and the quite large number of independent variables, regression analyses were performed using the composite scores for adaptive skills and language skills. Due to the high correlation (*r* = 0.80, *p* < 0.001) between language skills and social communication, we evaluated each predictor separately.

## Results

Table 2 shows the statistics for the independent variables and the outcome of maladaptive behavior.

**Table 2:** Summary of independent variables and the outcome variable (maladaptive behavior).

**Variables**	***N*** = **61**
Vineland-II scales (standard score)	
*Daily living skills* M (SD)	41.3 (16.64)
*Social skills* M (SD)	35.32 (18.92)
*Motor skills* M (SD)	62.46 (17.82)
Maladaptive behavior index (V-scale)	
*V-scale* M (SD)	18.07 (2.59)
*Elevated score* (≥18)%	41
*Clinically significant score* (21–24)%	18
Language and social communication	
*Sign language comprehension (adapted RDLS-III) assessed directly M (SD) (range 0–62)*	39.95 (13.65)
*Sign language comprehension (adapted CDI) reported by caregivers M (SD) (range 0–50)*	37.5 (12.27)
*Expressive language level (adapted PMLP) M (SD) (range 0–8)*	4.70 (2.00)
*Receptive language level (adapted PMLP)M (SD) (range 0–8)*	4.78 (2.04)
*Social communication (QSC-ID) M (SD) (range 1–5)*	2.83 (0.883)

*CDI, Child Development Inventory; PMLP, Profile of Multiple Language Proficiencies; RDLS-III, Reynell Developmental Language Scale-III; QSC-ID, social communication questionnaire in adults with intellectual disability*.

The mean for the maladaptive behavior index (v-scale) was 18.07 (SD = 2.59). Notably, 41% of the sample had elevated scores on the maladaptive behavior scale (i.e., v-scale scores ≥18), and a further 18% had clinically significant scores (i.e., v-scale scores between 21 and 24).

The assessment of the adaptive skills show a relative strength in motor skills with a mean standard score of 62.46, while the social skills are very low with a standard score of 35.32 and the daily living skills show a mean standard score of 41.3.

The results of the direct assessment of language comprehension by the adapted RDLS-III demonstrated severe deficits in the understanding of sign language. The use of English-based norms would result in a mean reference age of 3.51 indicating a large discrepancy between receptive language skills and non-verbal reference age (6.23). The highly corresponding proxy ratings of language comprehension resulted in an average reference age of 3.35 years by the use of English norms and confirmed the severity of language comprehension deficits as compared to the level of non-verbal intellectual functioning.

The mean results for expressive and receptive language levels (from 0 to 8) approximated by caregivers corresponded to the level of simple sentences (with four or more signs) and the ability to ask and answer basic questions. At this level, individuals have begun to use basic sign grammatical features. The level of expressive and receptive language was closely associated with the language comprehension skills measured by the RDLS-II and CDI.

Mean results for the QSC-ID (ranging from no to full command/implementation; 1–5) indicated limited skills, with relative strengths in the communication with caregivers, starting conversations, and sense of humor, and severe deficits in the prosocial use of language or non-verbal communication (e.g., use of compliments/appreciation and offering assistance).

The bivariate correlations between the maladaptive behavior index and the independent variables are shown in Table 3. The smallest (and non-significant) correlation was found for non-verbal cognitive functioning (*r* = −0.271). The correlations between maladaptive behavior and the Vineland-II Scales were somewhat larger, and for social skills (*r* = −0.272, *p* < 0.05) and the composite score (*r* = −0.276, *p* < 0.05) also significant. The largest correlations were found for language skills and social communication, ranging from *r* = −0.232 (*p* > 0.05) for receptive language skills measured by the RDLS-II to *r* = −0.405 (*p* < 0.001) for the CDI score.

**Table 3:** Bivariate correlations between the independent variables and the total maladaptive behavior score.

**Variable**	**Correlation with total maladaptive behavior**
Non-verbal cognitive functioning reference age	−0.217
Vineland adaptive skills	
*Social skills*	−0.272*
*Daily living skills*	−0.234
*Motor skills*	−0.219
*Vineland composite*	−0.276*
Language skills and social communication	
*Language skills (composite)*	−0.361**
*Social communication*	−0.378**
*Language skills and social communication (composite)*	−0.377**

**p <0.01, *p <0.05.

The regression models to predict total maladaptive behavior are shown in Table 4. In short, neither non-verbal cognitive functioning nor the Vineland-II adaptive skills composite score significantly predicted maladaptive behavior (Model 1 and Model 2). However, language skills and social communication were significantly associated with maladaptive behavior even after controlling for non-verbal cognitive functioning and Vineland-II adaptive skills (Model 3b).

**Table 4:** Regression models for the maladaptive behavior index.

	**Model 1**	**Model 2**	**Model 3a**	**Model 3b**

	β	β	β	β
Non-verbal cognitive functioning reference age	−0.220	−0.078	0.133	−0.025
Vineland adaptive skills		−0.230	−0.110	−0.093
Language skills and social communication				
*Language skills (composite)*			−0.387*	
*Social communication*				−0.317*
*R^2^*	0.048	0.081	0.141	0.151

*p <0.05. β standardized regression coefficient.

## Discussion

This study showed that in a sample of participants with deafness and special needs (mainly intellectual disability), the mean score for maladaptive behavior is elevated compared to the standardization sample. More specifically, within this highly vulnerable group, there was a prevalence rate of elevated maladaptive behavior of 41% and a clinically significant score in 18% of the participants. This rate is highly elevated despite the participants living in an environment that had been adapted to their needs for constant visual communication. This rate is also much higher than the general prevalence rate of challenging behavior in people with ID of 10–15% (18, 19). However, it should be noted that there might be differences between the definitions of maladaptive and challenging behavior used, as well as different instruments across studies, which makes a direct comparison between study groups difficult.

Language delays (in sign language) were found to be significantly more pronounced as compared to the participant's levels of intellectual and adaptive functioning.

We demonstrated that language skills and social communication explain an additional 14% of the variance in maladaptive behavior, while non-verbal cognition and adaptive behavior only explain 8% of the variance in a population of deaf people with special needs. This confirms our hypothesis that language and communicative skills have a stronger influence on maladaptive behavior than other factors. This is in line with previous studies (19) that found communication problems to be a common correlate of challenging behavior in people with ID. Furthermore, studies in children have shown an association between social communication and conduct disorder (27, 28).

In contrast to other studies on people with ID, we did not find a strong association between the level of intellectual functioning and maladaptive behavior. This underscores the role of communicative functioning in maladaptive behavior, at least in this specific group of individuals with severe communication difficulties. The large gap of almost 3 years between the mean cognitive reference age and the estimated reference age for language shows that the presence of early, severe language deprivation could not be overcome even in the highly adapted living environment. Due to a lack of measures of language deprivation in the current study and an expected lack of variance (from our knowledge of the participants' biographies all of them grew up with severe language deprivation) our data cannot verify the impact of language deprivation on maladaptive behavior in adulthood. However, by showing that intellectual functioning and adaptive skills did not have a large influence, we assume that it is the late access to accessible language that leads to persistent language and communication impairment associated with maladaptive behavior.

Our results emphasize the importance of early access to language in whatever mode and the constant promotion of communicative skills, as those people with better language and social communication skills demonstrated lower levels of maladaptive behavior.

The screening instrument for social communication (QSC-ID) has proven to be a useful tool to assess social-communicative abilities. Further research should investigate the practicability of the screening tool in longitudinal studies to measure increments of communicative abilities and give more insights into possible influences on the severity of maladaptive behavior. Furthermore, it would be interesting to look at the trajectory of social communication in a larger sample of individuals with ID and we predict that this would not be linear. We strongly recommend the use of measurements of language deprivation in further studies.

### Limitations

Due to the strong correlation between the adaptive skills composite and the social communication questionnaire, it was not possible to simultaneously evaluate both variables in predicting maladaptive behavior. Problems associated with collinearity are even more amplified given the quite small sample size of the current study. Related to the sample size, the study also had quite a low power, which makes it difficult for small to moderate effects to become significant. Finally, the analyses were based on cross-sectional data, making it impossible to make inferences about any causal associations between maladaptive behavior and the independent variables. The nature of the highly adapted environment offering constant visual communication in which the participants of this study had been recruited means we cannot generalize our results to other populations of people who are deaf and have special needs. All the participants had experienced severe language deprivation. Due to the lack of detailled data on variance of language deprivation we could not include this variable into our model to predict maladaptive behavior.

## Conclusion

The study findings illustrate the important influences that language and social communication have on maladaptive behavior. Findings also highlight the need to assess social communication as well as to foster social communication development in all people with special needs, independent of their cognitive functioning.

## Data availability statement

The datasets presented in this article are not readily available because the participants or their legal guardians did not consent to make the data publicly available. Requests to access the datasets should be directed to MD, magdalena.dall@jku.at.

## Ethics statement

The studies involving human participants were reviewed and approved by Ethical Committee of the Konventhospital Barmherzige Brüder Linz. The ethical approval number is EK 14-08. Written informed consent to participate in this study was provided by the participants' legal guardian/next of kin.

## Author contributions

JF, MD, and DH: conceptualization and investigation. CW: formal analysis. JF, MD, CW, and DH: writing—original draft preparation and writing—review and editing. All authors contributed to the article and approved the submitted version.

## Conflict of interest

The authors declare that the research was conducted in the absence of any commercial or financial relationships that could be construed as a potential conflict of interest.

## Publisher's note

All claims expressed in this article are solely those of the authors and do not necessarily represent those of their affiliated organizations, or those of the publisher, the editors and the reviewers. Any product that may be evaluated in this article, or claim that may be made by its manufacturer, is not guaranteed or endorsed by the publisher.
